# Effects of elevated atmospheric ozone concentration on biomass and non-structural carbohydrates allocation of cherry radish

**DOI:** 10.3389/fpls.2025.1547359

**Published:** 2025-02-20

**Authors:** Li Li, Bingkai Yang, Jinling Li, Xiaoke Wang, Sami Ullah

**Affiliations:** ^1^ Co-Innovation Center for Sustainable Forestry in Southern China, Bamboo Research Institute, Nanjing Forestry University, Nanjing, Jiangsu, China; ^2^ Forestry School, Guangxi Eco-engineering Vocational and Technical College, Liuzhou, Guangxi, China; ^3^ State Key Laboratory of Urban and Regional Ecology, Research Center for Eco-Environmental Sciences, Chinese Academy of Sciences, Beijing, China; ^4^ School of Geography, Earth and Environmental Sciences & Birmingham Institute of Forest Research, University of Birmingham, Birmingham, United Kingdom

**Keywords:** O3 pollution, chlorophyll contents, nonstructural carbohydrates, yield, root vegetable

## Abstract

Regional increases in atmospheric O_3_ have phytotoxicity due to its strong oxidizing properties. Cherry radish (*Raphanus sativus* L.), with its sensitivity to O_3_ and rapid growth cycle, serves as an excellent model for investigating the effects of elevated O_3_ on plant physiological responses. To determine the response of cherry radish to elevated O_3_ levels, we used nine open-top chambers with three O_3_ concentrations (Ambient-LO; 70 ppb O_3_ above ambient-MO; 140 ppb O_3_ above ambient−HO) in Beijing, China to examine the MDA, chlorophyll contents, biomass, soluble sugar, and starch contents in response to O_3_ exposure. The results showed that: 1) elevated O_3_ (EO_3_) did not affect leaf chlorophyll contents but increased carotenoid contents; (2). The total biomass, hypocotyl biomass and hypocotyl size were significantly decreased by 41% and 49%, 51% and 37%, 53% and 40% by MO and HO, respectively. The above-to-below-ground biomass ratio (A/B) increased by 49% and 61% under MO and HO treatments; (3). HO and MO significantly increased leaf fructose, sucrose, and glucose contents by 192% and 79%, 40% and 37%, 110% and 45%, respectively; (4). leaf soluble sugar biomass proportion increased by 75% and 99% under HO and MO mainly contributed by fructose biomass proportion increase; (5). radish plant allocated more soluble sugar, starch and NSC biomass proportion to leaf but not hypocotyl under EO_3_. In conclusion, radishes exposed to O_3_ allocate more nonstructural carbohydrates (NSC) to the leaf at the expense of a great loss of hypocotyl biomass. This is possible mainly due to compensation of O_3_-induced damage via the sugar transport pathways, where transport is blocked so that the inefficient conversion of soluble sugars into starch can lead to reduced biomass accumulation and ultimately lower crop yields of radish. The role of radish fructose in protecting against or responding to O_3_ risks may be underestimated as it affects the overall sugar metabolism and transport within the plant.

## 1 Introductions

Tropospheric ozone (O_3_) is an oxidative, phytotoxic air pollutant that threatens crop production and agricultural security (Ramya et al., 2023). O_3_ forms in the lower atmosphere through complex photochemical reactions involving nitrogen oxides (NOx) and volatile organic compounds (VOCs), both emitted by industrial activities, transportation, and natural sources. Globally, O_3_ concentrations have been increasing in some of the world’s metropolitan areas, with urban population exposure to O_3_ increasing at 89% of stations (+0.8% yr^-1^), such as Equatorial Africa, South Korea, India and China, due to lower O_3_ titration by NO (Sicard et al., 2023).

Mean annual O_3_ concentrations (maximum 8-hour day) in 74 major cities in China have been reported to increase by 28.8% from 2013 to 2019 (CAOPPBB, 2020) due to persistently high emissions of the primary precursors of O_3_ (volatile organic compounds and nitrogen oxides) (Li et al., 2021). The oxidizing nature of O_3_ is responsible for its phytotoxic effects on crops. O_3_ enters plants through stomata and stimulates the overproduction of reactive oxygen species (ROS) (Tiwari et al., 2018), which target membrane permeability via lipid peroxidation of the bilipid layer of membranes (Tripathi and Agrawal, 2013). Lipid peroxidation is commonly measured as malonaldehyde (MDA) content and has been correlated with the degree of membrane damage under O_3_ exposure, and is therefore often used as a biomarker of oxidative stress (Li et al., 2024).

The chlorophyll content plays an important role in determining the photosynthetic yield of plants, while the reduction of chlorophyll content upon O_3_ treatment is well documented in the literature (Li et al., 2015; Choquette et al., 2020). O_3_ could also affect the chlorophyll ratio. Pellegrini (2014) observed a decrease in the ratio of total chlorophyll to carotenoids, implying an enhancement of photoprotective de-excitation pathways mediated by carotenoids of plants (Pellegrini, 2014). Exposure of crops to high O_3_ concentrations is a major threat to global food security as it would lead to significant yield losses by impairing photosynthesis (Bisbis et al., 2018; Ramya et al., 2023; Leung et al., 2022). Feng et al. (2019) reported that elevated tropospheric O_3_ concentrations in China reduced annual rice and wheat yields by 8% and 6%, respectively, in 2015. The cost of O_3_-induced losses in 2015 was ~$7.5 billion for rice and $11.1 billion for wheat. O_3_ has induced losses in major crops (Mills et al., 2011) such as wheat, rice (Feng et al., 2019) and common bean, sorghum (Sorghum bicolor), pearl millet, finger millet (Sharps et al., 2021) and radish (Manning, 2023) due to impairment of photosynthesis. Apart from biomass reduction, O_3_ stress also leads to changes in photosynthate C allocation, with more biomass being used for O_3_ injury repairs rather than being converted to storage starch in different plant parts (Li et al., 2022).

Carbohydrates produced by photosynthesis in plants are mainly stored in the form of sugars and starches, which constitute nonstructural carbohydrates (NSCs) for various metabolic activities (Bansal and Germino, 2009; Hoch, 2015). Soluble sugars including fructose, sucrose, and glucose, serve as a direct source of energy, while starch primarily functions as a rapid nutrient storage form (Li et al., 2022). It is reported that EO_3_ can increase soluble sugar and alter sugar distribution among organs for osmotic adjustment, scavenging of reactive oxygen species, and signaling, while starch decreases to produce soluble sugar to enhance photosynthetic activity and defense mechanisms in the leaves (Tiwari et al., 2018).

Photosynthetic partitioning changes under EO_3_, particularly concerning carbohydrate allocation between aboveground and belowground in many species, thereby affecting the balance between structural and nonstructural carbohydrates (NSC) (Chen et al., 2018). Pools of NSCs in different plant organs can serve as buffers to counteract decreases in C assimilation in response to environmental stressors such as O_3_ exposure (Li et al., 2022) and the imbalance between C supply and demand across plant tissues (Du et al., 2020). The components of NSCs, starch and soluble sugars (sucrose, fructose, glucose), play different roles in modifying O_3_ stress (AbdElgawad et al., 2020), and their ratio may reflect the strategy for regulating a sustainable carbon balance between organs. Therefore, the concentration, accumulation, and proportional allocation of NSCs in different organs could reflect the status of carbon supply in the whole plant, providing insights into the strategy and ability of plants to grow and survive in the face of environmental perturbations. O_3_ could affect the allocation of non-structural carbohydrates, which has been reported in wheat (Sild et al., 2002) and soybean (Zheng et al., 2019), but we found it rare in root vegetables such as radish.

Cherry radish (*Raphanus sativus* L.) is a common root vegetable valued for its bright red color, crisp texture and fast growth. It’s widely used in salads and various culinary dishes for its refreshing taste and nutritional benefits. In China, the area under radish cultivation is up to 1.2 million square hectares, with an annual production of more than 40 million tons (Xing et al., 2024). Cherry radish has been studied for responses to root temperature and O_3_ (Kleier et al., 2001), cultivar-specific O_3_ sensitivity (Hassan et al., 2018), and gas exchange, growth, and nutrient status to CO_2_ and O_3_ (Barnes and Pfirrmann, 1992). In this study, we planted cheery radish in Changping, Beijing, China. The patterns of leaf MDA, pigments, biomass and NSC content and allocation were well-studied using open-top chambers (OTCs). We study how EO_3_ influences above and below-ground biomass and NSC allocation and how the relative components of NSC (soluble sugar and starch) of cherry radish are affected. Our hypotheses are: 1) EO_3_ concentrations will reduce biomass accumulation to reduce both above- and below-ground biomass due to chlorophyll reduction; 2) EO_3_ concentrations will increase sugar of above ground for resistance to O_3_ stress and decrease starch content of all plant parts for rapid nutrient supply.

## 2 Materials and methods

### 2.1 Experimental site and plant material

The experimental site was in Chang Ping district, a suburb of Beijing, China (40°12′N, 116°80′E). Beijing has a typical temperate and monsoonal climate with four clearly distinct seasons. The annual mean precipitation of the experimental site was 550.3 mm and the mean air temperature was 11.°C. The local soil in the experimental site is generally characterized as moist with moderate fertility (Tong et al., 2012), and therefore provided the required conditions to promote the growth of radish. The soil used in the pots contained an organic content of 16.4 g/kg, a total nitrogen content of 0.9 g/kg, an available phosphorus content of 102.1mg/kg, and a pH of 8.3. All plants were watered as needed and fertilized once with 4 g of specialized carbamide and potassium sulfate fertilizer (N14:P14: K14, Wuhan Greenovo Biotechnology Co., Ltd., China) prior to the experiment. A common radish variety planted in the fall (*Raphanus sativus* L. var. radculus Pers.) was selected, and 216 seeds were planted evenly into 36 pots (six seeds per pot) on August 27th. We used 5 kg soil in each pot (top diameter: 27.5 cm, base diameter: 18.5 cm, height: 21 cm), then 6 pots were randomly distributed to 1 of the 6 open-top chambers (OTCs).

This radish variety has a growing period of 30-45 days, is characterized by highly productive, drought-resistant, and low-temperature resistance, and is relatively common in autumn plantings in Beijing. The seedlings per pot were thinned to 4 and then moved to the ambient chambers to be accustomed to the environment until Sep. 15th.

### 2.2 OTCs

The OTCs were the same as in Li et al. (2022). The OTCs were made of an aluminum alloy frame covered with transparent plastic film with a 10-mm thickness (90% transparency). More details on OTC design, operation, O_3_ concentration control, and distribution control can also be found in Li et al. (2015) and Li et al. (2022). O_3_ concentrations were evenly distributed, both vertically and horizontally, within the OTCs. The Model 49i O_3_ analyzer (Thermo Scientific, MA, USA) was calibrated every month with a Model 49i-PS O_3_ analyzer (Thermo Scientific, MA, USA). A 10% variation in O_3_ concentrations around the target O_3_ concentrations was allowed in this experiment.

### 2.3 Experimental design and sampling

**Figure 1:**
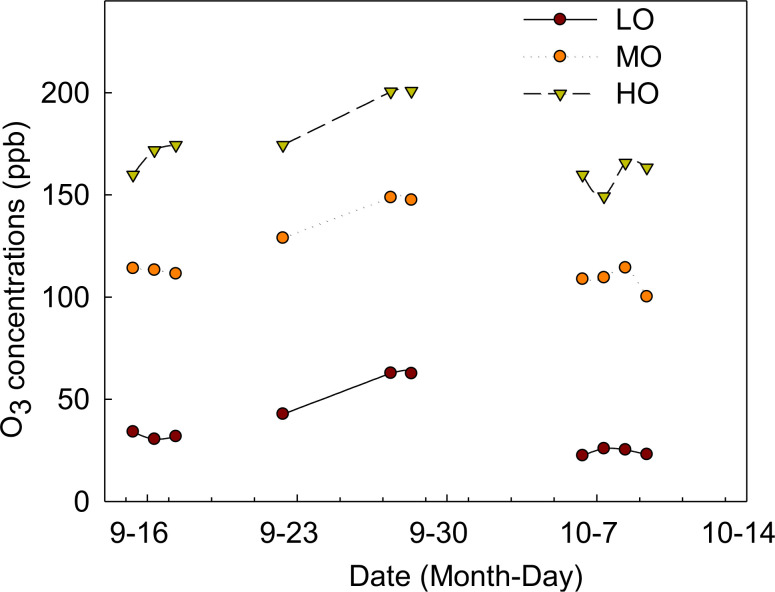
O_3_ exposure during the experiment LO means low O_3_ treatments; MO means medium O_3_ treatments; HO means high O_3_ treatments.

Three O_3_ treatments were randomly assigned to nine open-top chambers (OTCs). Due to the increasing O_3_ concentrations in Beijing (Wang et al., 2012; BMEPB, 2015; Li et al., 2020, 2021), the O_3_ concentrations in the present study were selected to match the levels in ambient air (low O_3_-LO), in addition to ambient air + 70 ppb O_3_ (medium O_3_-MO) and ambient air +140 ppb O_3_ (high O_3_-HO). O_3_ exposure started on September 15th and ended on October 10th. The duration was determined by the growth period of the radish. O_3_ was applied through fumigation from 8:30 to 17:30 (9 h) each day. The average ambient air (AA) O_3_ concentration in the experimental sites was 36.1 ppb, ranging from 22.4 ppb to 62.8 ppb throughout the day during the experimental periods (**Figure 1**). The average O_3_ concentrations of AA+70 and AA+140 were 119.7 ppb and 172.0 ppb (**Figure 1**), respectively, meaning that the O_3_ control could meet the target requirements. Due to the strong winds around October 7th, the O_3_ concentrations of HO treatments had a small fluctuation, but the mean value can still meet our target concentrations (**Figure 1**). The O_3_ was applied daily except for rainy days, for a total of 10 days of O_3_ exposure during the experimental period (**Figure 1**). The pots with the same treatments were interchanged to different positions of each chamber or different chambers with the same treatments every other day. Plants including green leaves were harvested at the end of the experiment for biomass, MDA, chlorophyll and NSC measurements.

### 2.4 Measurements

Three mature green leaves were collected from three radishes per treatment at the end of the experiment for MDA and chlorophyll measurements after biomass measurements. Radish plants were separated as leaf and hypocotyl where the leaves were taken as aboveground biomass and the edible hypocotyl was taken as “blowground”. The rest attached root was not counted in this experiment because it was very tiny and took only around 3% of the total biomass.

Leaf was collected and stored in 4°C incubators with ice bags then transferred to the -80°C fridge. Leaf MDA contents were determined by microplate spectrometric assays as described by Wang et al. (2014). 50 mg of leaf tissue per plant was extracted with 4 ml 95% ethanol in the dark for 48 h at room temperature. The absorbance of leaf pigment extracts was measured at 646, 450 and 663 nm using a spectrophotometer (UV2101PC, Shimadzu, Kyoto, Japan) for chlorophyll a (Chla), chlorophyll b (Chlb) and carotene (Car). Chlorophyll contents were calculated according to the specific absorption coefficients provided by Lichtenthaler (1987). The total chlorophyll content was taken as the sum of Chla, Chlb, and Car.

Biomass (separated as aboveground and belowground parts) was determined by drying up at 70°C to a constant weight prior to analysis. The biomass samples of all parts were then finely ground to powder with mortar and liquid nitrogen for NSC measurements. NSC contents (soluble sugar and starch contents) were determined as described by Li et al. (2022) with slight modifications. For soluble sugar components, we have measured fructose, sucrose, and glucose contents. Sucrose was determined by accurately aspirating 10ml of extract into a test tube, adding 2ml of 2mol/L KOH followed by boiling in water for 10 minutes, taking out and quickly cool down to room temperature (put in cold water), pour into a 50ml volumetric flask, and then finally set the volume with distilled water. Accurately aspirate 2ml of the dilution solution into a clean test tube, add 6ml of anthrone reagent, shake well, immediately place in a boiling water bath and heat for 5 minutes, remove and immediately cool rapidly in cold water with constant shaking. Read the absorbance value at 640nm and check the standard curve to get the amount of soluble sugar. Glucose, fructose, sucrose, and starch were determined by the anthrone-sulfuric acid method (Ma et al., 2021). Starch content (mg·g^-1^) was determined as described by Grantz and Yang (2000). NSC allocation proportions were calculated using the ratio of the products of target NSC components contents and target biomass to the whole plant NSC mass (Li et al., 2022).

### 2.5 Statistical analyses

The effects of O_3_ on all parameters were examined by one-way ANOVA. *Post-hoc* comparisons were conducted using the Duncan test. Prior to analysis, all data were checked for normality (Kolmogorov-Smirnov test) and homogeneity of variance (Levene’s test). If the data were not normally distributed and/or their variance was not homogeneous, non-parametric tests were used. Results were considered significant when *P*<0.05. All analyses were performed using the SPSS statistics software (Version 17.0, SPSS Inc., Chicago, IL, USA).

## 3 Results

### 3.1 Malondialdehyde

**Figure 2:**
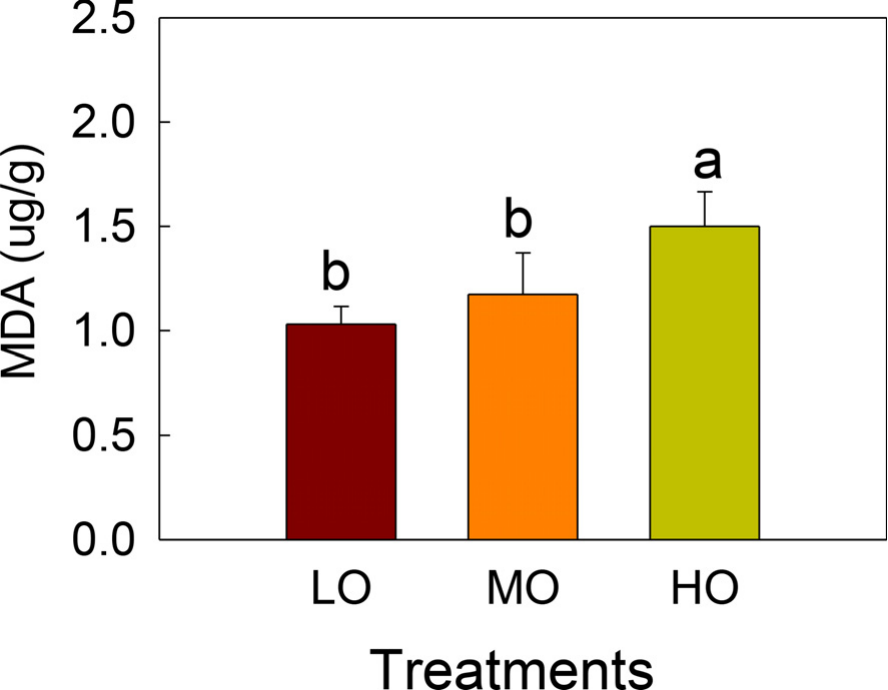
The effects of elevated O_3_ on leaf MDA contents of radish Values are represented as mean ± SE. Different lowercase letters above bars indicate significant multiple comparisons between treatments when *P*<0.05. LO means low O_3_ treatments; MO means medium O_3_ treatments; HO means high O_3_ treatments.

MDA contents were only influenced by high O_3_ (HO) treatments, which showed a 49% increase compared with the control. Medium O_3_ treatment (MO) did not show significant differences with the control treatment (LO) (**Figure 2**).

### 3.2 Chlorophyll contents

**Figure 3:**
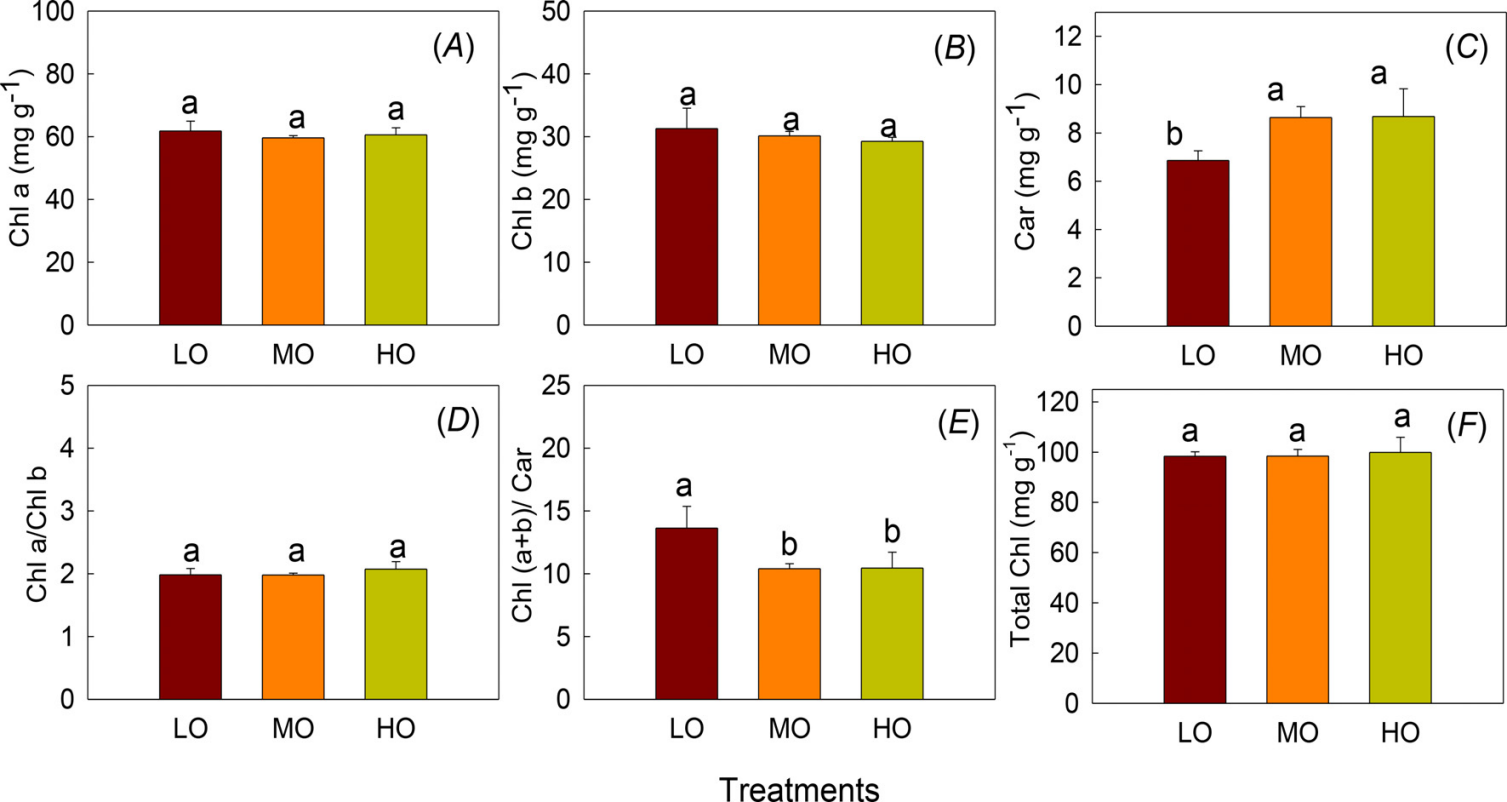
The effects of elevated O_3_ on chlorophyll content (Chl a, **A**), chlorophyll b contents (Chl b, **B**) and carotenoids (Car, **C**), Chl a/Chl b **(D)**, Chl (a+b)/Car **(E)** and total chlorophyll (total Chl, **F**) Values are represented as mean ± SE. Different lowercase letters above bars indicate significant multiple comparisons between treatments when *P*<0.05. LO means low O_3_ treatments; MO means medium O_3_ treatments; HO means high O_3_ treatments.

Chla, Chlb and Chla/Chlb were not affected by EO_3_ treatments while Car was increased by 26.1% and 26.2% by MO and HO, respectively (**Figure 3**). However, HO and MO decreased Ch(a+b)/Car by 23.3% and 23.7% compared with LO. (**Figure 3E**). No differences between MO and HO were detected (**Figure 3**).

### 3.3 Biomass

**Figure 4:**
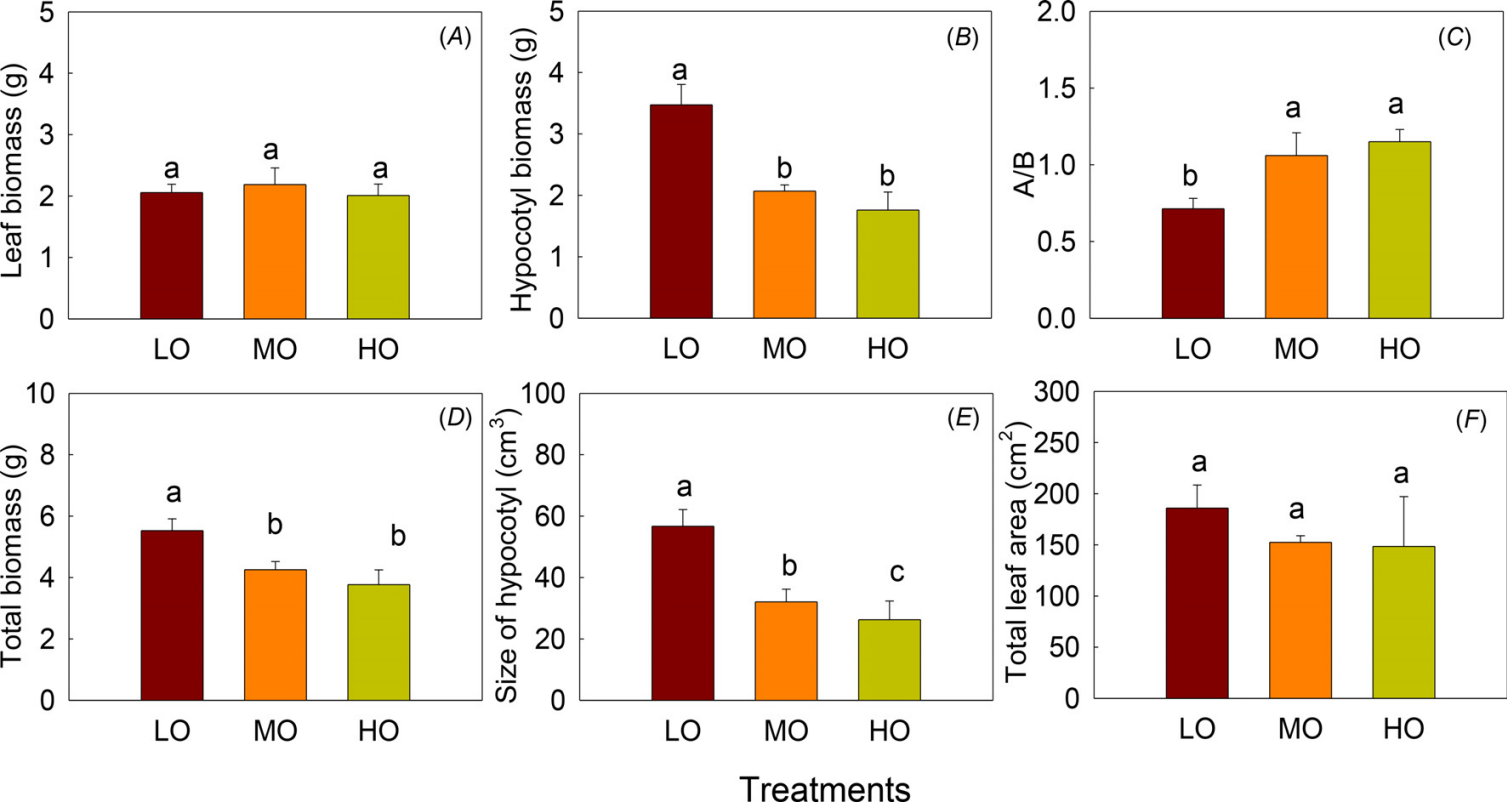
The effects of elevated O_3_ on leaf biomass **(A)**, hypocotyl biomass **(B)**, ratio of above-ground to below-ground biomass **(A/B, C)**, total biomass **(D)**, size of hypocotyl **(E)** and the total leaf area **(F)** Values are represented as mean ± SE. Different lowercase letters above bars indicate significant multiple comparisons between treatments when *P*<0.05. LO means low O_3_ treatments; MO means medium O_3_ treatments; HO means high O_3_ treatments.

The results indicated that EO_3_ did not affect leaf biomass and total leaf area but significantly decreased hypocotyl biomass and hypocotyl size (*P*<0.05) (**Figures 4A, B, D, E, F**). The total biomass, hypocotyl biomass and hypocotyl size were significantly decreased by 41% and 49%, 51% and 37%, 53% and 40% by MO and HO, respectively. The above-to-below-ground biomass ratio (A/B) increased by 49% and 61% under MO and HO treatments (**Figure 4C**). Hypocotyl biomass, A/B, and the total biomass did not show differences between MO and HO (**Figures 4B–D**).

### 3.4 NSC contents

#### 3.4.1 Leaf NSC contents

**Figure 5:**
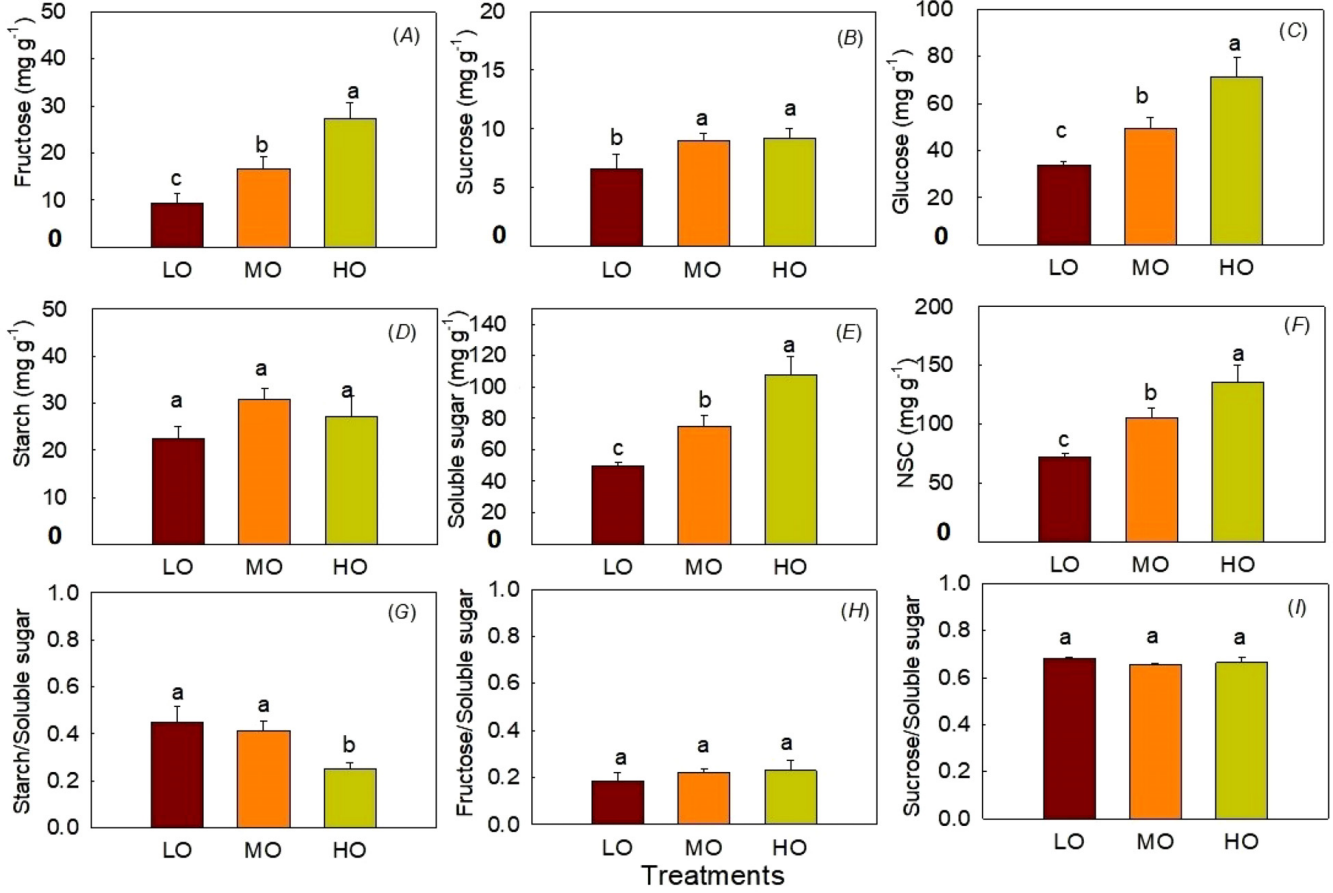
Effects of elevated O_3_ on leaf fructose contents **(A)**, sucrose contents **(B)**, and glucose contents **(C)**, starch contents **(D)**, soluble sugar contents **(E)**, NSC contents **(F)**, starch to soluble sugar ratio **(G)**, fructose to soluble sugar ratio **(H)** and sucrose to soluble sugar ratio **(I)** Values are represented as mean ± SE. Different lowercase letters above bars indicate significant multiple comparisons between treatments when *P*<0.05. LO means low O_3_ treatments; MO means medium O_3_ treatments; HO means high O_3_ treatments.

The results showed HO and MO significantly increased fructose, sucrose, glucose, soluble sugar and NSC contents by 192% and 79% (**Figure 5A**), 40% and 37% (**Figure 5B**), 110% and 45% (**Figure 5C**), 117% and 50% (**Figure 5E**), 87% and 46% (**Figure 5F**). The ratio of starch to soluble sugar significantly decreased 44% by HO (**Figure 5G**). HO and MO showed differences between fructose, glucose contents, soluble sugar, NSC and the ratio of starch to soluble sugar (**Figure 5G**). EO_3_ did not affect leaf starch contents (**Figure 5D**). The ratio of leaf fructose and sucrose to soluble sugar was not influenced by O_3_ treatments (**Figures 5H, I**).

#### 3.4.2 Hypocotyl NSC contents

**Figure 6:**
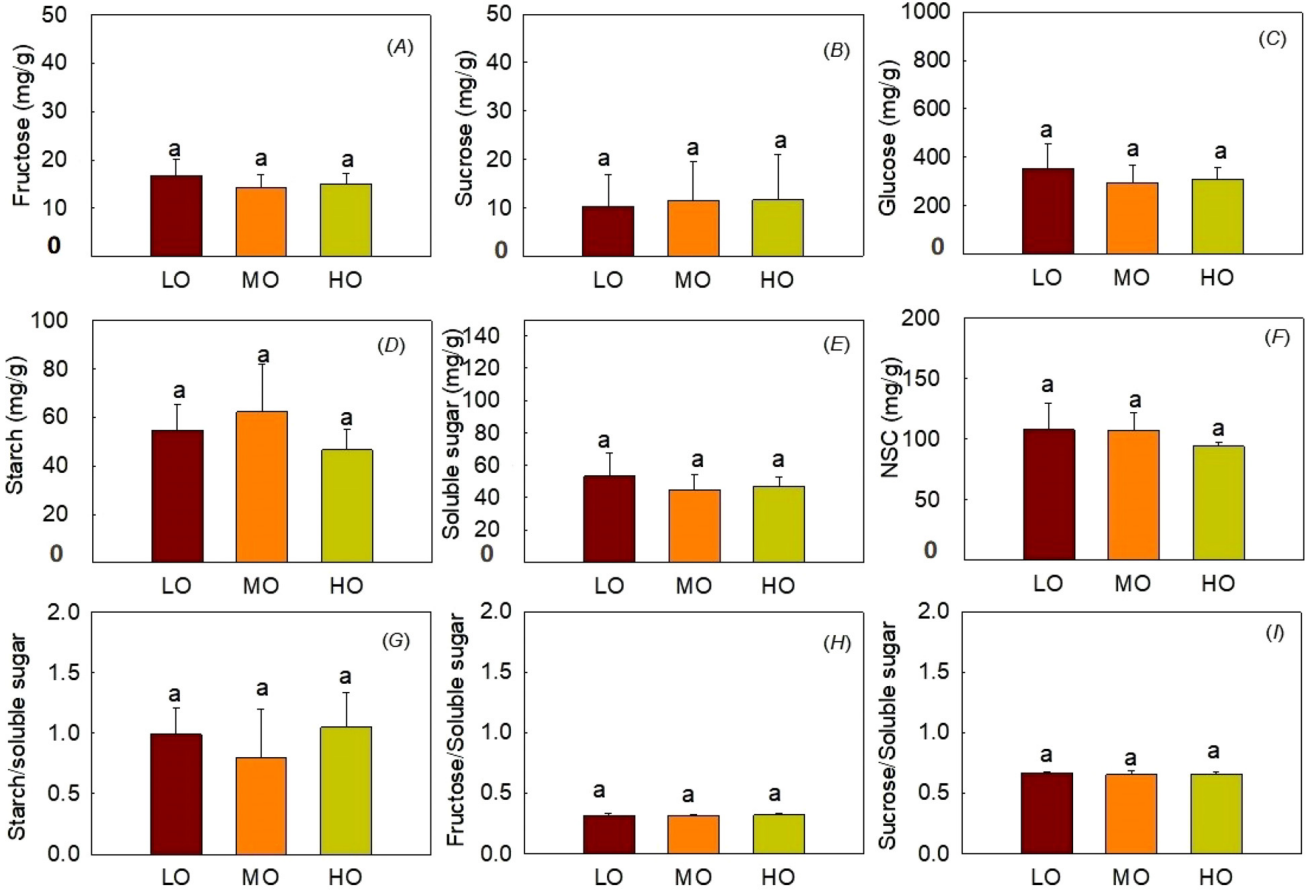
Effects of elevated O_3_ on hypocotyl fructose contents**(A)**, sucrose contents **(B)**, and glucose contents **(C)**, starch contents **(D)**, soluble sugar contents **(E)**, NSC contents **(F)**, starch to soluble sugar ratio **(G)**, fructose to soluble sugar ratio **(H)** and sucrose to soluble sugar ratio **(I)** Values are represented as mean ± SE. Different lowercase letters above bars indicate significant multiple comparisons between treatments when *P*<0.05. LO means low O_3_ treatments; MO means medium O_3_ treatments; HO means high O_3_ treatments.

The results indicated that EO_3_ did not affect fructose contents, sucrose contents, glucose contents, starch contents, soluble sugar contents, NSC contents and the ratio of starch to soluble sugar (**Figure 6**). The ratio of hypocotyl fructose and sucrose to soluble sugar was not influenced by O_3_ treatments (**Figures 6H, I**).

### 3.5 NSC allocation proportions

#### 3.5.1 Soluble sugar, starch, and NSC allocation proportions

**Figure 7:**
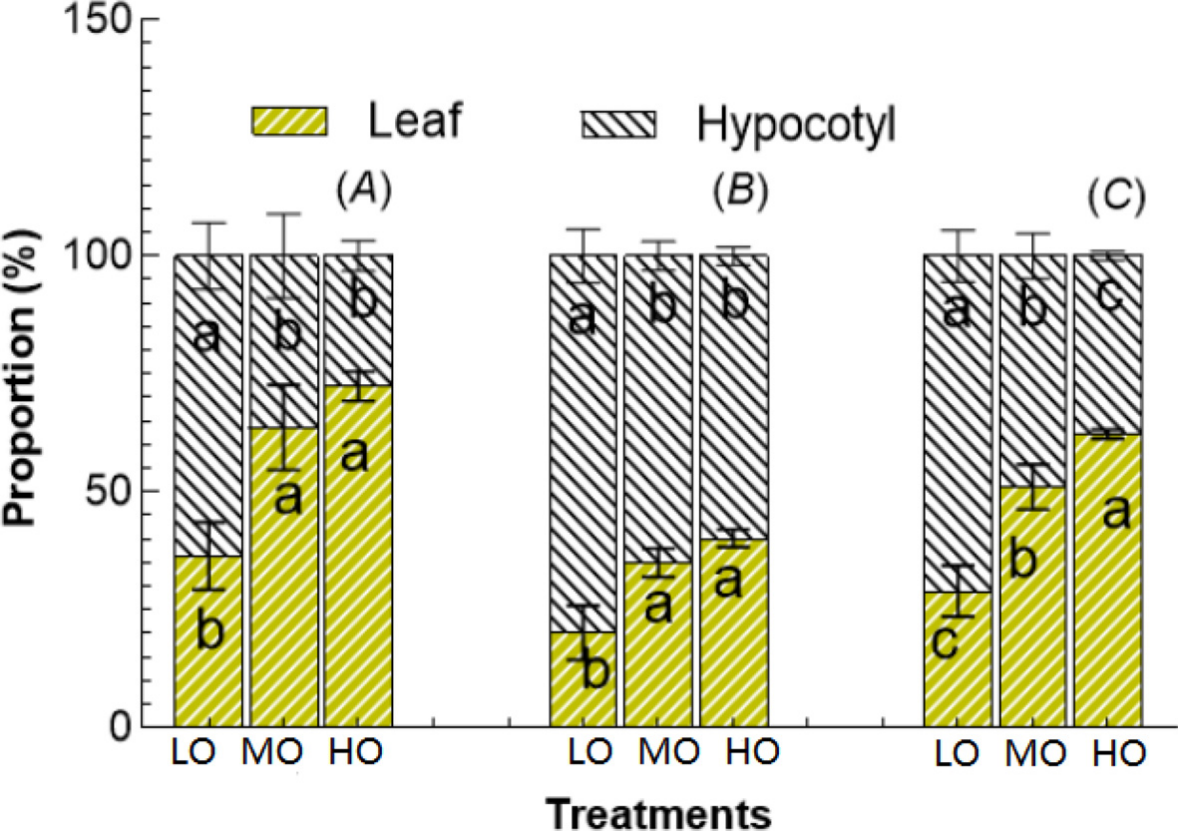
The proportion of soluble sugar **(A)**, starch **(B)**, and NSC **(C)** between leaf and hypocotyl under three O_3_ concentration treatments. Values are represented as mean ± SE. Different lowercase letters inside bars indicate significant multiple comparisons between treatments when *P*<0.05. LO means low O_3_ treatments; MO means medium O_3_ treatments; HO means high O_3_ treatments.

The results indicated that the HO and MO significantly increased the allocation and proportion to leaf soluble sugar by 75% and 99% (**Figure 7A**), to leaf starch by 74% and 99% (**Figure 7B**) and to NSC by 76% and 115% (**Figure 7C**) while reduced the allocation proportion to hypocotyl soluble sugar by 57% and 43% (**Figure 7A**), starch by 25% and 19% (**Figure 7B**), NSC by 47% and 31%, respectively (**Figure 7C**).

#### 3.5.2 Leaf and hypocotyl soluble sugar allocation proportions

**Figure 8:**
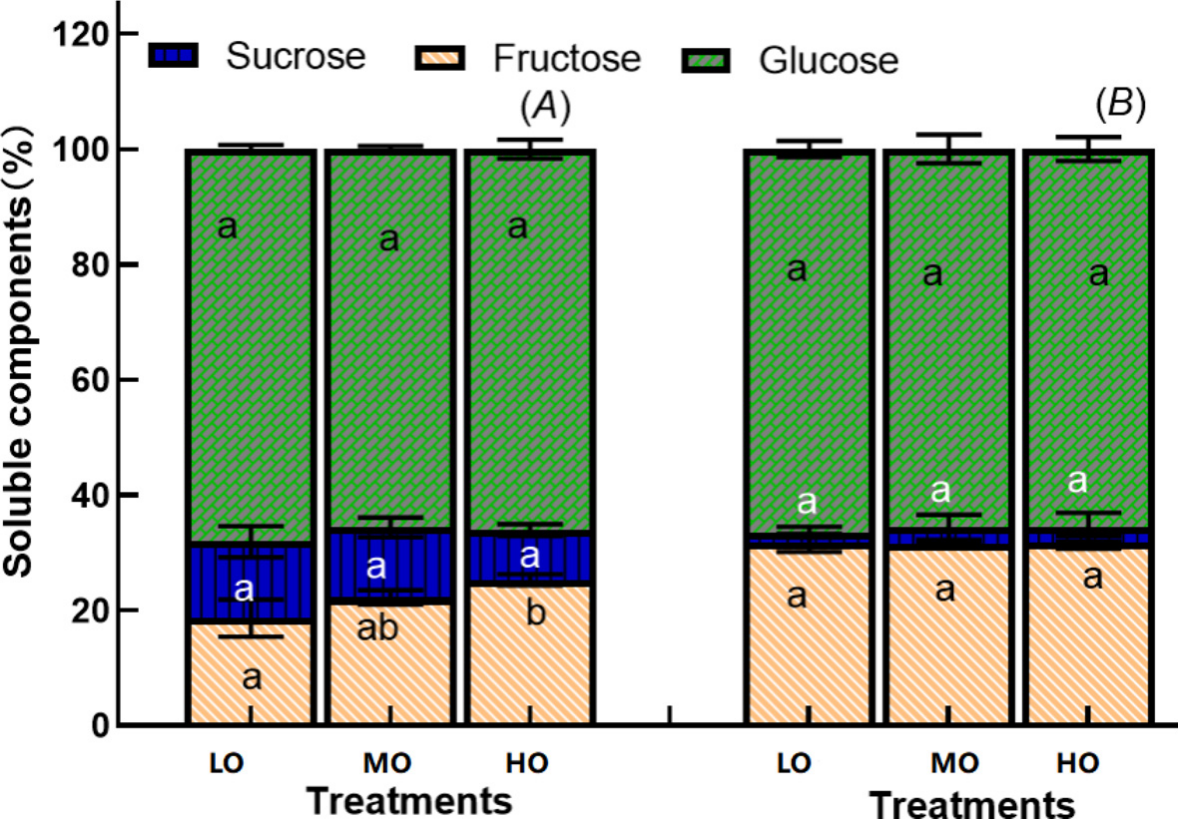
Effects of elevated O_3_ on soluble sugar components proportion of leaf **(A)** and hypocotyl **(B)** Values are represented as mean ± SE. Different lowercase letters inside bars indicate significant multiple comparisons between treatments when *P*<0.05. LO means low O_3_ treatments; MO means medium O_3_ treatments; HO means high O_3_ treatments.

Although EO_3_ significantly increases the leaf fructose, sucrose, glucose contents and soluble sugar proportion (**Figures 6**, **7**), the enhancement of leaf soluble sugar of leaf proportion of radish plant is mainly due to the increased fructose proportion (**Figure 8A**). The results indicated that HO and MO increased the fructose proportion by 36% and 19% (**Figure 8A**) while the sucrose and glucose were not affected by EO_3_. The proportions of hypocotyl were not affected by treatments (**Figure 8B**).

## 4 Discussion

### 4.1 MDA and chlorophyll contents

Lipid peroxidation is generally measured as malonaldehyde (MDA) content and has been correlated with the degree of membrane damage under O_3_ exposure (Li et al., 2024). We found that leaf MDA contents increased significantly by 46% only under HO (≈160ppb) after 20 days of fumigation, but not under MO (**Figure 1**), implying that EO_3_ could lead to a decrease in membrane stability and an increase in membrane damage in cherry radish leaves. It has been reported that MDA levels increase when reactive oxygen species (ROS), such as singlet oxygen and hydroxyl radicals, attack the unsaturated lipids present in cell membranes under oxidative stress such as O_3_ (Chaudhary and Rathore, 2021). Our result also suggests that the cherry radish cultivar selected in this experiment may have a higher O_3_ tolerance capacity, as Tiwari and Agrawal (2011) found that the MDA content of radish (Raphnus sativus L cv Pusa Reshmi) leaves increased significantly after 60 days when grown even at a mean O_3_ concentration of 40.8 ppb (8h).

Chlorophyll is the primary pigment involved in capturing light energy for photosynthesis (Croce and van Amerongen, 2020). Although O_3_-induced oxidative stress has been reported to damage chlorophyll molecules and disrupt photosynthesis (Li et al., 2015), we found that chlorophyll content remains unchanged in mature leaves (**Figure 3**). The damage caused by EO_3_ depends on both plant development stage, O_3_ dose, intensity and duration of O_3_ fumigation (Ainsworth and Rogers, 2007; Li et al., 2022). The fact that the chlorophyll content of mature radish leaves remains unchanged may be due to effective protective mechanisms and the strategy to maintain photosynthetic efficiency at this stage of radish growth (Pell et al., 1997), as indicated by the unchanged leaf biomass (**Figure 4**), which may involve repair mechanisms for damaged chlorophyll (Castagna and Ranieri, 2009). Regulating chlorophyll levels to ensure continued photosynthesis despite stress is critical, as a reduction in chlorophyll content would reduce the plant’s ability to capture light energy, further affecting growth and survival, and thus leading to reduced biomass accumulation. Sakalauskaitė et al. (2008) also reported that EO_3_ did not have a significant negative effect on photosynthetic pigment synthesis in radishes. The unchanged chlorophyll results indicated that the acute O_3_ exposure in this experiment was still within a range where the defense mechanisms of radish could cope and chlorophyll levels could remain stable.

The results showed that carotenoids were increased by both HO and MO (**Figure 3**). The explanation is that leaf carotenoids, a class of pigmented compounds synthesized by plants play a crucial role in photoprotection by scavenging reactive oxygen species (ROS) and absorbing excess light energy, especially under stress conditions (Pellegrini et al., 2015) or by their involvement in non-photochemical quenching of Chl fluorescence (Casper-Lindley and Björkman, 1998). EO_3_ induces plants to enhance their antioxidant defense, including the synthesis of carotenoids (Singh et al., 2023). The ratio of total chlorophyll to carotenoids decreases at the end of fumigation (**Figure 3**), which is considered an early stress indicator (Tiwari et al., 2018), suggesting that plants need to invest in the enhancement of carotenoid-mediated photoprotective quenching pathways (Pellegrini, 2014; Tiwari et al., 2018). Although we have not found similar research on radish, this result of increased Chl/Car ratio is consistent with the findings of Pellegrini et al. (2011) in Salvia officinalis, which confirmed that O_3_-treated leaves increased the need for carotenoid-mediated photoprotection.

### 4.2 Biomass and NSCs

NSC accumulation in source tissues (leaves) and their transport to sink tissues (underground parts, fruits) regulates overall crop productivity and quality (Xu et al., 2024). During stress conditions, such as nutrient deficiency or environmental stress (e.g., elevated O_3_), NSC allocation can shift from growth to maintenance, reducing biomass production and yield (Li et al., 2022; Xu et al., 2024). Radish exposed to EO_3_ showed lower A/B rates, meaning that the O_3_-induced reduction in hypocotyl biomass was greater than the reduction in shoot growth (above ground) (**Figure 4**), which was in contradiction to our first hypothesis. The possible reasons why leaf biomass remains relatively unaffected while hypocotyl growth is inhibited are compensatory responses. Leaves are crucial for photosynthesis, the main energy source of the plant. As an annual crucifer, radish under EO_3_ may prioritize resource allocation to maintain critical functions (photosynthesis, reproduction) according to the optimal allocation hypothesis (Kozłowski, 1992). As a compensatory response, the inhibited belowground biomass of radish implies a reallocation of carbon resources away from hypocotyl growth processes towards leaf photosynthesis, defense mechanisms, or reproduction generation, as reported in potatoes (Asensi-Fabado et al., 2010). Therefore, plants may divert resources to leaf protection at the expense of reduced hypocotyl growth, biomass, and NSC partitioning.

O_3_ stress causes significant changes in the metabolite contents of plants (Gandin et al., 2021). Consistent with the biomass results, leaf soluble sugar content increased under EO_3_, and the proportions of leaf soluble sugar and starch also increased (**Figures 4**, **5**, **7**), but hypocotyl starch content was not affected by EO_3_ treatments, which is partially different from the second hypothesis. The possible reasons are: 1. EO_3_ directly damages chloroplasts and reduces photosynthetic rates (Li et al., 2022), so the increase in soluble sugars reflects an attempt to maintain energy flux, repair injury, and adjust carbohydrate metabolism through the O_3_-induced source-sink imbalance; 2. hypocotyl starch content was not affected by EO_3_, which may be due to phloem transport with consequent inhibition of translocation to belowground parts, as reported by Grantz and Young (2000). This inhibition may lead to the accumulation of photosynthates such as fructose in leaves instead of being transported to other parts of the plant (below ground) or used for growth and development (Li et al., 2018); 3. Changes in sugar metabolism can activate defense mechanisms. Soluble sugars, such as glucose and fructose, can act as signaling molecules that induce the expression of defense-related genes (Papazian and Blande, 2020). EO_3_ could affect plant metabolism, including the production and accumulation of certain sugars of non-structural carbohydrates such as fructose, as our results show (**Figure 8**). Fructose, as a means of maintaining cellular homeostasis, has been reported to protect cellular structures from oxidative damage and to maintain osmotic balance (Sanità di Toppi and Gabbrielli, 2021); 4. Increased levels of soluble sugars could also increase respiratory activity, as these sugars serve as primary substrates with the necessary energy for cellular respiration (Rashid et al., 2020); 5. Sugars, including fructose, act as signaling molecules in plants, regulating gene expression related to stress responses. EO_3_ could trigger an increase in sugar signaling to initiate protective mechanisms, leading to an accumulation of these soluble sugars (Koch, 2019). However, changes in sugar composition, with increased levels of soluble sugars, particularly fructose, will attract more insects to feed, which may increase the risk of yield loss (Xia et al., 2021). Furthermore, the harvest time in this experiment of this radish is in the reproductive phase (50 days), and the increase in sugar levels may indicate that more energy is being allocated to the leaf for regeneration early on, as EO_3_ can also induce leaf senescence (van Doorn, 2008). Sugar accumulates in leaves and leads to carbon imbalance, which induces sugar phosphorylation and down-regulates of the photosynthesis (Piccolo et al., 2020). Fructose and glucose showed the highest increase among the soluble sugar components (**Figure 8**). This is because sucrose, as a photosynthetic end product, must be hydrolyzed to glucose and fructose by invertase or catalyzed to fructose by sucrose synthase before it can be used (Ruan, 2014). Glucose and fructose are then used for growth as well as stored and transported, as facilitated by sugar transporters (Ruan, 2014). The decrease in the ratio of leaf starch to soluble sugars (**Figure 5**) is part of a complex cascade of effects triggered by O_3_ stress, including disrupting the balance of carbon allocation within the plant which may lead to altered source-sink relationships (Gupta et al., 2015). Starch-to-soluble sugar ratio under EO_3_ is indicative of complex adjustments in plant metabolism aimed at coping with environmental stress, which may reflect or determine plant health and productivity (Sánchez-Rodríguez et al., 2012). A lower starch-to-soluble sugar ratio (**Figure 5**) could be a plant strategy to cope with O_3_-induced oxidative stress because soluble sugars, such as glucose and fructose, can act as antioxidants and mitigate cell damage (Polle et al., 2023). However, a lower ratio of starch to soluble sugars in the leaf results in less transfer of photosynthate production to the other organs, as the leaf acts as a source of photosynthates (such as starch and sugars) that are transported to other parts of the plant (sinks), such as fruits, seeds or roots (hypocotyl), where they are used or stored. The inefficient conversion of soluble sugars into starch can lead to reduced biomass accumulation and ultimately lower crop yields (Melash et al., 2023).

The proportion of more starch and more soluble sugars from hypocotyl to leaf (**Figure 7**) reflects that O_3_ could cause shifts in the partitioning of assimilates from storage compounds (i.e. starch) to compounds (i.e. soluble sugars) involved in the O_3_ injury repair response (Li et al., 2022). Under stress, plants often reallocate resources to prioritize survival and defense over growth. This is a ‘trade-off’ between promoting some metabolic functions at the expense of reducing growth to develop the production of defense metabolites (Matyssek and Sandermann, 2003; Grantz et al., 2006). This can further affect the energy balance of the plant and may lead to a situation where more energy is consumed by respiration than is produced by photosynthesis (Feng et al., 2008), negatively affecting plant fitness. The higher proportion of fructose in leaves under EO_3_ (**Figure 8**) means that EO_3_-induced starch hydrolysis into simpler sugars, particularly fructose, by the oxidative stress to provide energy for repair mechanisms or to act as antioxidants (Thalmann and Santelia, 2017). Cherry radish is a very common green vegetable that is easily threatened by EO_3_. However, very few studies have investigated the adverse effects of O_3_ pollution on radish through the accumulation and variation of non-structural carbohydrate (NSC) allocation and its implication for production. Our study provides quantitative insights into the effects of O_3_ on MDA, chlorophyll, biomass and NSC, indicating that EO_3_ increases more soluble sugars, especially fructose and carotenoids in the aboveground plant parts (leaves) rather than in the below-ground edible part, causing a large loss in the hypocotyl. Radish exposed to O_3_ allocates more nonstructural carbohydrates to the leaf at the expense of a great loss of hypocotyl biomass. Globally, O_3_ concentrations have been increasing in some of the world’s metropolitan areas as one of the major air pollutants, the effects of O_3_ on root crops may be underestimated due to a whole biomass assessment approach, which would further underestimate the loss of the edible part belowground.

## Data Availability

The raw data supporting the conclusions of this article will be made available by the authors, without undue reservation.
